# Genomic diversity of citrate fermentation in *Klebsiella pneumoniae*

**DOI:** 10.1186/1471-2180-9-168

**Published:** 2009-08-15

**Authors:** Ying-Tsong Chen, Tsai-Lien Liao, Keh-Ming Wu, Tsai-Ling Lauderdale, Jing-Jou Yan, I-Wen Huang, Min-Chi Lu, Yi-Chyi Lai, Yen-Ming Liu, Hung-Yu Shu, Jin-Town Wang, Ih-Jen Su, Shih-Feng Tsai

**Affiliations:** 1 Division of Molecular and Genomic Medicine, National Health Research Institutes, Miaoli, Taiwan, Republic of China; 2 Genome Research Center and Institute of Biomedical Informatics, National Yang-Ming University, Taipei, Taiwan, Republic of China; 3 Division of Infectious Diseases, National Health Research Institutes, Zhunan, Miaoli, Taiwan, Republic of China; 4 Department of Pathology, National Cheng Kung University Hospital, Tainan, Taiwan, Republic of China; 5 College of Medicine, Chung Shan Medical University, Taichung, Taiwan, Republic of China; 6 Department of Bioscience Technology, Chang Jung Christian University, Tainan County, Taiwan, Republic of China; 7 Department and Graduate Institute of Microbiology, National Taiwan University, Taipei, Taiwan, Republic of China; 8 Department of Life Sciences and Institute of Genome Sciences, National Yang-Ming University, Taipei, Taiwan, Republic of China

## Abstract

**Background:**

It has long been recognized that *Klebsiella pneumoniae *can grow anaerobically on citrate. Genes responsible for citrate fermentation of *K. pneumoniae *were known to be located in a 13-kb gene cluster on the chromosome. By whole genome comparison of the available *K. pneumoniae *sequences (MGH 78578, 342, and NTUH-K2044), however, we discovered that the fermentation gene cluster was present in MGH 78578 and 342, but absent in NTUH-K2044. In the present study, the previously unknown genome diversity of citrate fermentation among *K. pneumoniae *clinical isolates was investigated.

**Results:**

Using a genomic microarray containing probe sequences from multiple *K. pneumoniae *strains, we investigated genetic diversity among *K. pneumoniae *clinical isolates and found that a genomic region containing the citrate fermentation genes was not universally present in all strains. We confirmed by PCR analysis that the gene cluster was detectable in about half of the strains tested. To demonstrate the metabolic function of the genomic region, anaerobic growth of *K. pneumoniae *in artificial urine medium (AUM) was examined for ten strains with different clinical histories and genomic backgrounds, and the citrate fermentation potential was found correlated with the genomic region. PCR detection of the genomic region yielded high positive rates among a variety of clinical isolates collected from urine, blood, wound infection, and pneumonia. Conserved genetic organizations in the vicinity of the citrate fermentation gene clusters among *K. pneumoniae*, *Salmonella enterica*, and *Escherichia coli *suggest that the13-kb genomic region were not independently acquired.

**Conclusion:**

Not all, but nearly half of the *K. pneumoniae *clinical isolates carry the genes responsible for anaerobic growth on citrate. Genomic variation of citrate fermentation genes in *K. pneumoniae *may contribute to metabolic diversity and adaptation to variable nutrient conditions in different environments.

## Background

Citrate, a ubiquitous natural compound that exists in all living cells, can be used by several enterobacterial species as a carbon and energy source. *Klebsiella pneumoniae *has been known to be able to grow anaerobically with citrate as the sole carbon source. During the past decade, the physiology, biochemistry, and regulation of this pathway have been extensively studied in *K. pneumoniae *[1-4]. The fermentation process involves uptake of citrate by a Na+ -dependent citrate carrier, cleavage into oxaloacetate and acetate by citrate lyase, and decarboxylation of oxaloacetate to pyruvate by oxaloacetate decarboxylase. Finally, pyruvate can be converted to acetate, formate and carbon dioxide by means of anaerobic pyruvate catabolism.

Genes responsible for citrate fermentation of *K. pneumoniae *can be identified in a 13-kb gene cluster on the chromosome [[2,5], and this study]. These genes are contained within two divergently transcribed operons, *citC2D2E2F2G2 *and *citS-oadGAB-citAB *[6]. The *citC2D2E2F2G2 *operon encodes the citrate lyase ligase, the γ-, β-, and α-subunits of citrate lyase, and triphosphoribosyl-dephospho-coenzyme A synthase. The *citS-oadGAB(dcoCAB)-citAB *operon encodes the citrate carrier CitS, the γ-, α-, and β-subunits of oxaloacetate decarboxylase, and the citrate-sensing CitA-CitB two component system [5]. Transcription at the promoters in front of the two operons is activated by phospho-CitB and Crp-cAMP [2]. Additionally, *citX*, which is required for synthesis of the citrate lyase prosthetic group, has been identified in a second genomic location along with *citW*, a putative citrate transporter gene, and *citYZ *that encodes a two component system homologous to CitA-CitB [7]. The *citWX *genes and the divergent *citYZ *are adjacent but placed in opposite directions.

Coliform organisms, especially *E. coli *and *K. pneumoniae*, are the most common causes of urinary tract infection. Uropathogenic pathogens have been studied extensively for virulence factors such as the fimbriae and adhesins [8,9]. These virulence factors facilitate the anchorage of the pathogens to the extracellular matrix of the bladder and urinary tract, and thus prevent them from being washed out by the urine. Type I pili, which is produced by all members of the *Enterobacteriaceae *family, has long been implicated as an important virulence factor in mediating *K. pneumoniae *urinary infection [10,11]. Alternatively, the ability to grow in urine may be important for the persistence of pathogens in the urinary tract. Except for trace of amino acids, citrate is the only carbon source available in normal human urine.

In *K. pneumoniae*, little has been reported about the genomic basis for nutrient growth. We recently completed the whole-genome sequence of NTUH-K2044 (GenBank accession no. AP006725) [12], a *K. pneumoniae *strain isolated from the blood of a previously healthy individual who was diagnosed with a community-acquired primary liver abscess and metastatic meningitis [13]. By comparison with the available genome sequences of the other *K. pneumoniae *strains, MGH 78578 (GenBank: CP000647), and 342 (GenBank: CP000964) [14], we discovered that the entire 13-kb chromosomal region carrying the aforementioned citrate fermentation genes in MGH 78578 and 342 was missing in NTUH-K2044. We postulated that the 13-kb genomic region containing genes for citrate fermentation might facilitate the use of urine citrate in oxygen-limited or anaerobic conditions, and thus, permit the growth of *K. pneumoniae *in the urinary tract. To test this hypothesis, an artificial urine medium (AUM) designed to provide controlled composition of the human urine [15] was used in this study to ensure reproducibility. The correlation between presence/absence of the citrate fermentation genes and anaerobic growth in this system was investigated. The distribution of the citrate fermentation genes among different *K. pneumoniae *clinical isolates was also analyzed.

## Results and Discussion

### The citrate fermentation genes in a 13-kb genomic region

**Figure 1:**
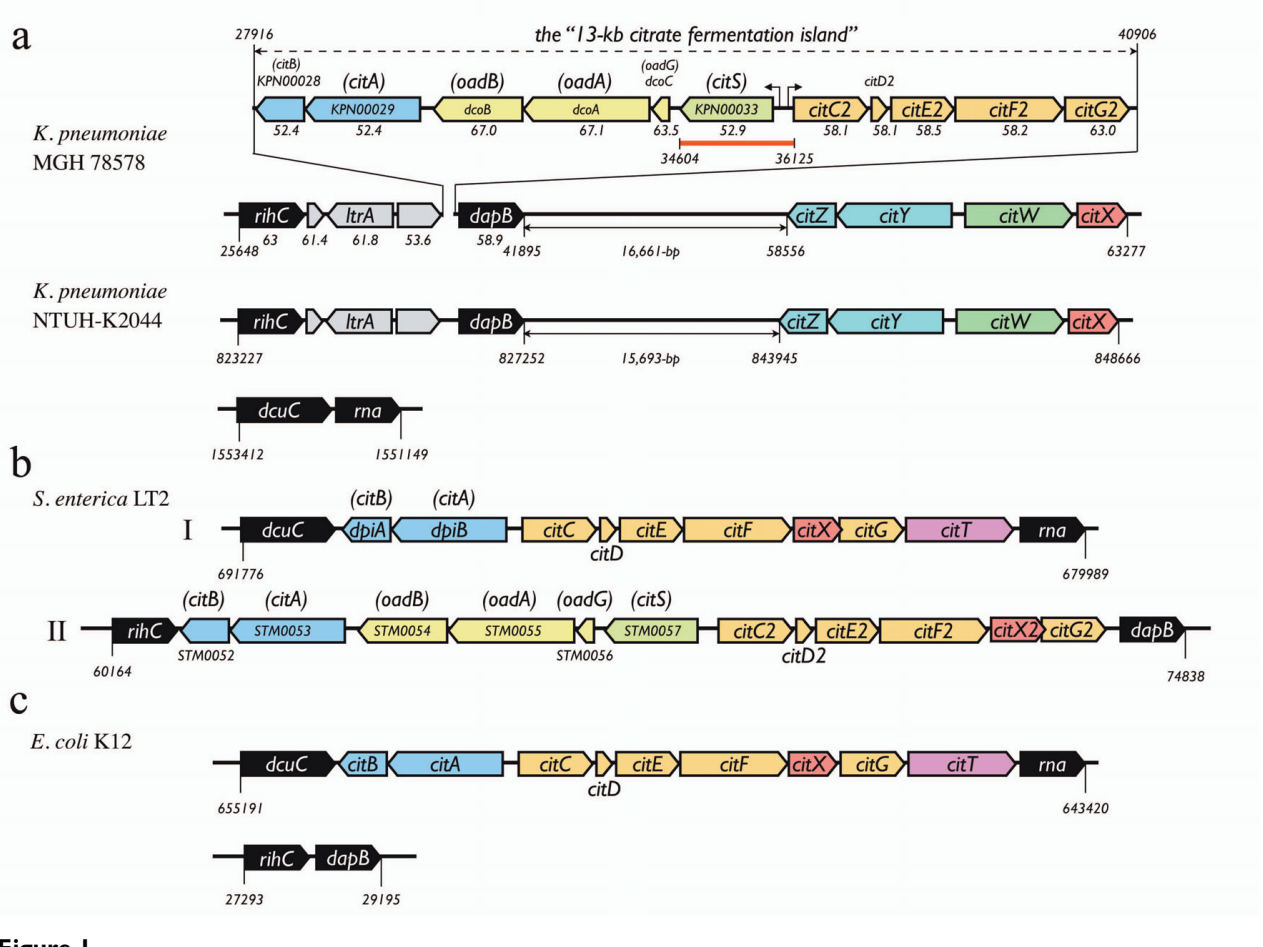
**Comparative analysis of citrate fermentation gene locus**. The 13-kb genomic region is present in *K*. *pneumoniae *MGH 78578 but absent in NTUH-K2044 (a). The location of the 13-kb genomic region for citrate fermentation, which includes two divergently transcribed operons, *citS*-*oadGAB*-*citAB *and *citC2D2E2F2G2*, are marked. The adjacent hypothetical orfs are shown in gray, among which the *ltrA *encodes a putative transcriptional regulator. The *citYZ-citWX *gene clusters downstream of the *dapB *in both MGH 78578 and NTUH-K2044 are also depicted. The G+C value for each orf in MGH 78578 is shown below each orf. The red bar indicates the corresponding location replaced by an apramycin resistant gene in the promoter knocked-out strain, NK8-Δcit, derived from the NK8 clinical strain. Corresponding citrate fermentation loci from *S. enterica *serovar Typhimurium LT2 and *E. coli *K12 are shown (b and c) with colours indicating homologous genes. Alternative gene names in parentheses on top of some orfs for better comparison were based on homology search. The locations of these regions in the genomes are marked below. In the LT2 genome, two clusters of citrate fermentation genes were found. The corresponding flanking genes for locus I, *dcuC *and *rna*, and locus II, *rihC *and *dapB*, are shown in black.

Located at 27916-40906 bp in the genomic sequence of *K. pneumoniae *strain MGH 78578, the 13-kb citrate fermentation gene locus contains 11 orfs, which constitute two divergently transcribed operons *citC2D2E2F2G2 *and *citS-oadGAB(dcoCAB)-citAB *(Figure 1). The organization of these genes is the same as in the recently published *K. pneumoniae *342 genome [14]. The dihydrodipicolinate reductase gene *dapB *and the hypothetical orfs located at the two ends of the 13-kb region in the MGH 78578 and 342 genomes are next to each other in the NTUH-K2044 genome. Missing in the corresponding location, the citrate genes are nowhere found in the NTUH-K2044 genome, and the region is replaced by a 155-bp non-coding sequence. Since many genomic or pathogenicity islands found in bacteria genomes were associated with tRNA genes, we also tried to look for tRNA genes at the edge of this region. However, it appeared that the 13-kb genomic region carrying the citrate fermentation genes is not located within or near any tRNA gene, nor does it contain any direct repeat or known mobility sequence. This is in agreement with a recent study of bacterial genome flux, which indicated that, among twenty *Escherichia coli *genomes, many of the integration hotspots are not necessarily recombinogenic [16].

Another gene cluster containing the *citWX *and the divergent *citYZ *genes are conserved among *K. pneumoniae *genomes (Figure 1a). In NTUH-K2044, the *citWX-citYZ *gene cluster is located at 15,693-bp downstream of the *dapB*. The existence of this additional gene cluster, especially the *citX*, is important for the function of citrate lyase in *K. pneumoniae*. Unlike the counterpart identified in *Salmonella enterica *(Figure 1b), the 13-kb region in *K. pneumoniae *does not contain *citX *for the biosynthesis of the prosthetic group of citrate lyase [7]. In MGH 78578, the deduced amino acid sequences of *citY *and *citZ *are 43% and 41% identical to CitA and CitB, respectively.

### Nearly half of the *K. pneumoniae *clinical isolates carry the 13-kb genomic island

**Figure 2:**
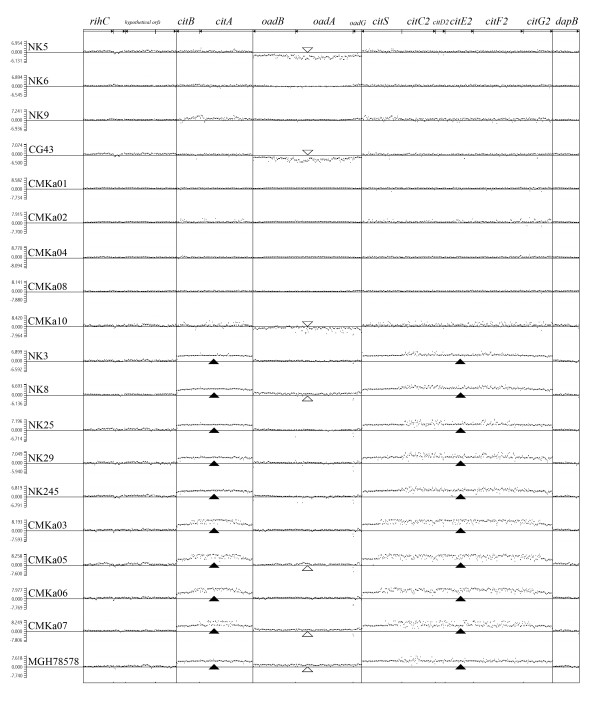
**Genomic variation at the citrate fermentation gene locus**. Divergence of the 13-kb genomic region in 19 *K*. *pneumoniae *strains was detected by CGH analysis using the NimbleGen chips. Hybridization signals of each probes placed in the order of the MGH 78578 genome were compared with those of the reference strain, NTUH-K2044. The probes covering the *cit *genes and the *oad *genes of the 13-kb region were shown together with that of the adjacent *orfs*. The normalized CGH signals for each probe are plotted as black dots. The dot position above or under the baseline represents higher or lower copy of specific genomic sequence in comparison to the reference. The scores in vertical axis are log2 values of test/reference signal intensity obtained from image scanning of hybridization results. The detection of elevated scores in the *cit *genes (*citA-B*, *citS~citG2*) in the last 10 strains (from NK3 to MGH 78278) is marked by solid triangles. Variations in the *oad *region are marked by open triangles.

The presence/absence of the 13-kb region was investigated in additional *K. pneumoniae *clinical isolates (NK3, NK5, NK6, NK8, NK9, NK25, NK29, NK245, CG43, CMKa01 through CMKa08, CMKa10). These isolates were collected from patients with pneumonia (3), bacteremia (4), liver abscess (7), UTI (2), meningitis (1), and endophthalmitis (1). We conducted comparative genomic hybridization (CGH) analysis on the test strains with custom-made DNA microarray (NimbleGen), in which a total of 389,266 probes were designed based on the CDSs of five sequenced *K. pneumoniae *genomes [12]. For the current report, we have analyzed the results of the predicted coding sequences spanning the 13-kb region of MGH 78578. As shown in Figure 2, each of the 19 strains (including MGH 78578 as a control) was compared against the NTUH-K2044 reference genome. The dots represent the DNA copy number log ratios between the reference and tested genomes for the 687 probes corresponding to the sequences spanning the 13-kb region. Since the NTUH-K2044 genome does not carry the *cit *genes, these results indicate that the 9 strains with dots plotted at the baseline in this region (NK5, NK6, NK9, CG43, CMKa01, CMKa02, CMKa04, CMKa08, and CMKa10) do not carry these genes in their genomes. The other ten strains shown in below, including MGH 78578, gave higher signals for the *cit *genes than that from the reference (Figure 2). By contrast, all strains have the same copy number of the flanking genes (*rihC, hypothetical orfs*, and *dapB*) as the NTUH-K2044.

The *oad *genes within the 13-kb region are missing in NTUH-K2044, but the strain possesses an additional copy of *oad *genes at the tartrate-fermentation gene cluster outside this region. In contrast, according to the genomic sequence, MGH 78578 (GenBank: CP000647) carries three copies of the *oad *genes, including one in the 13-kb region. This is also confirmed by the CGH result, which indicated that four strains, MGH 78578, NK8, CMKa05, and CMKa07, carry more than one copy of the *oad *genes and showed higher signal in the *oad*-probed region. On the other hand, CMKa10, NK5 and CG43, do not have *oad *genes and were represented by CGH plots below the baseline. We conclude that the 13-kb citrate fermentation gene sequence is not a uniform feature of *K. pneumoniae *and that the *oadGAB *gene copy number is variable among the analyzed strains.

In a recent report, it is shown that all *K. pneumoniae *strains could grow on citrate as sole carbon source when tested aerobically [17]. A stark contrast is the ability of *K. pneumoniae *to grown on citrate anaerobically. While all *K. pneumoniae *isolates can grow on citrate aerobically, our results suggested that only about half of them carry the 13-kb gene cluster for anaerobic citrate utilization.

### The 13-kb genomic island permits anaerobic growth in artificial urine

As citrate is a major carbon source in human urine, we then asked whether the 13-kb genomic island could contribute to *K. pneumoniae *growth in the urinary tract. Although human urine is a suitable culture medium, the urine constituents can vary considerably between individuals under different conditions. It has been reported that the dissolved oxygen (DO) in urine is about 4.2 ppm, which is also variable and mainly reflects the renal metabolic state [18]. In patients with urinary infections, the urine DO is significantly reduced as a result of oxygen consumption by the microbes [18]. Therefore, in this study an artificial urine medium (AUM) developed to provide an experimental condition similar to that of the human urine [15] was used. To simulate growth conditions in the urinary tract, *K. pneumoniae *isolates were cultured in AUM at 37° under oxygen-deprived condition.

**Figure 3:**
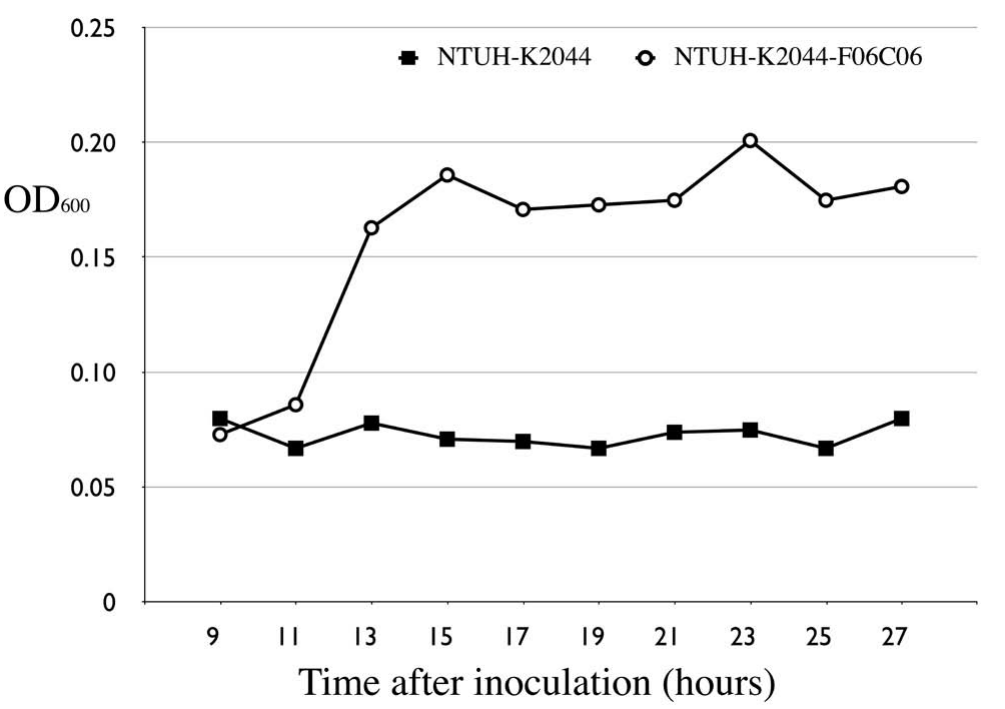
**Citrate gene cluster permits fermentation growth in AUM for the NTUH-K2044 strain**. NTUH-K2044, a strain that lacks the 13-kb genomic region; NTUH-K2044-F06C06, NTUH-K2044 transformed by a fosmid (F06C06) carrying the 13-kb genomic region responsible for citrate fermentation from NK8.

**Figure 4:**
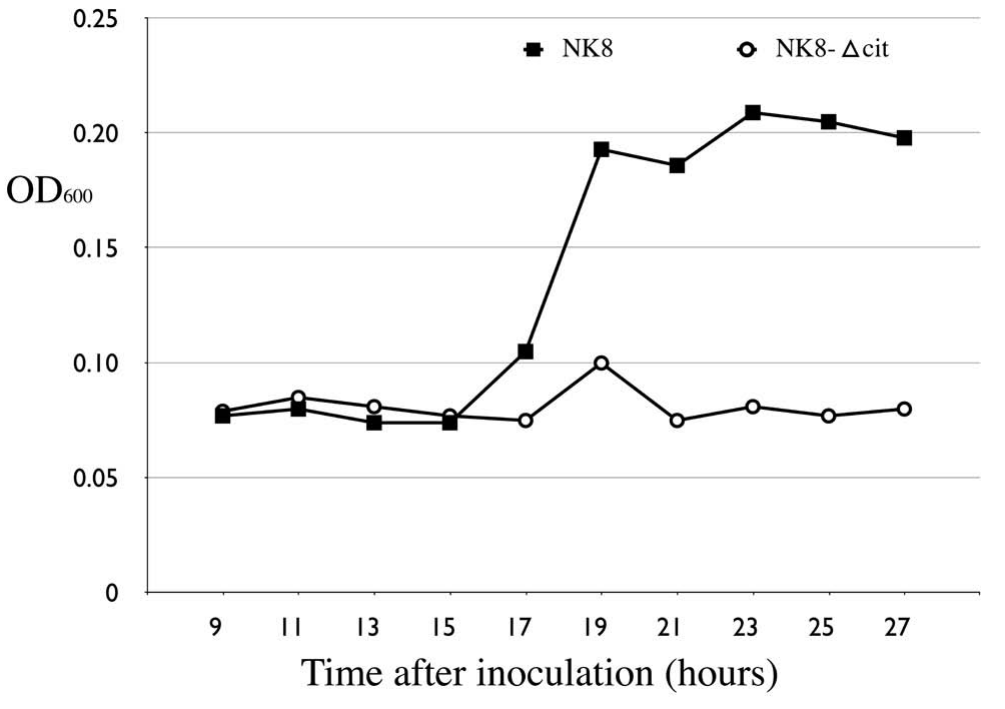
**Citrate gene cluster is necessary for fermentation growth in AUM for the NK8 strain**. NK8 is a clinical strain carrying the same citrate fermentation genes as the sequenced reference strain, MGH 78578; NK8-Δcit, NK8 with the 13-kb genomic region disrupted at the promoter region. The initial OD_600 _of the inoculated AUM culture is 0.0005.

Notable difference in the growth curves was observed when *K. pneumoniae *clinical strains were cultured anaerobically in AUM. After 27 hours incubation, five strains with the 13-kb genomic island (NK3, NK8, NK25, NK29, NK245), showed significant growth in AUM (OD_600_: 0.17-0.43). In contrast, little growth (OD_600_: 0.04-0.06) was detected for strains that do not have the 13-kb genomic island (NTUH-K2044, NK5, NK6, NK9, CG43). The turbidities (OD_600_) of NK8 and NTUH-K2044 at different time points during the 27-hour incubation in AUM were also measured. Note that little growth was detected in NTUH-K2044, a strain that lacks the citrate fermentation gene cluster (Figure 3), while exponential logarithmic phase growth was observed from 15 to 19 h in the NK8 strain that carries the 13-kb genomic island (Figure 4).

To demonstrate that the citrate fermentation genes present in the 13-kb region have allowed alternative use of carbon and energy source, a fosmid, F06C06, which contains the entire 13-kb region from NK8, was transformed into NTUH-K2044. As shown in Figure 3, this fosmid enabled the bacteria (NTUH-K2044-F06C06) to grow anaerobically in AUM. The logarithmic growth (from 11 to 15 h) of the fosmid-transformed clone was shifted to the left and the cells reached the stationary phase earlier than that of the NK8. This may be a result of gene copy number discrepancies between the fosmid transformants and NK8, or a result of other genetic factors specific to the NTUH-K2044 genome. Similarly, the F06C06 fosmid sequence enabled the anaerobic growth of *E. coli *epi300 (Epicenter Technologies, Madison, WI) transformants in AUM (data not shown). As a control, the *K. pneumoniae *strains NTUH-K2044, NK8, NTUH-K2044-F06C06, and NK8-Δcit were cultured anaerobically in AUM medium prepared without citrate, all four strains showed no sign of growth in 27 hours.

To demonstrate that an intact citrate gene cluster is necessary for anaerobic growth, we created by homologous recombination a genetic mutant in which the entire *citS *and the nearby *citC2 *promoter was replaced with an apramycin resistance gene. The *citS*-*citC2 *intergenic region contains binding sites for the response regulator CitB and cyclic AMP receptor protein (CRP), which mediates catabolic repression of citrate fermentation genes under anaerobic conditions [4]. The gene disruption was confirmed by PCR and sequencing of the region. The corresponding location of the altered sequence in the citrate fermentation island is indicated in Figure 1a. As consistent with the fact that the *citC2 *and *citS *promoters control the expression of the *citC2D2E2F2G2 *and *citS-oadGAB-citAB *operons, disruption of this regulatory region in the resultant strain, NK8-Δcit, crippled its ability to grow anaerobically in AUM (OD_600 _= 0.042 after 27-h incubation) (Figure 4). Taken together, our data support that the citrate fermentation island permits and is necessary for anaerobic growth of *K. pneumoniae *in AUM using citrate as the sole carbon source.

### Citrate fermentation gene cluster in *K. pneumoniae *clinical isolates

From the genetic studies on the citrate fermentation in AUM, it seems plausible that the ability of *K. pneumoniae *to grow in urine may provide the organism an added advantage in urinary tract infections (UTI), thus a higher percentage of citrate fermentation genomic island-positive *K. pneumoniae *strains would be expected in urine isolates than in non-urine isolates. To test this hypothesis, a total of 187 *K. pneumoniae *clinical isolates collected from urine and non-urine specimens including blood, respiratory tract, wound, bile, ear, eye, and IV catheters, were analyzed for the presence of the 13-kb island by using 5 PCR primer pairs designed across the region (Table 1). As shown in Table 2, 55 out of the 93 (59%) urine isolates carried the genomic island, while 53/94 (56.3%) of non-urine were test positive for the gene cluster. Thus, we did not find apparent correlation between the possession of the 13-kb genomic region and urinary tract infection in this case collection.

**Table 1:** Primer pairs used for detecting citrate fermentation genes.

Primer sequences		Genes covered	Product size (bp)
1.	5'-CCGGGCCTGAATATTAAACA-3'	*citA*, *citB*	952
	5'-CAACAGCAGTCGGAAAGTCA-3'		
2.	5'-GGATCTTCCGCTCCTTATCC-3'	*oadA*, *oadB*	890
	5'-GGAAGCCATGAAGATGGAGA-3'		
3.	5'-GCCCATCAGGATAGTTGGAA-3'	*citS*, *citC2*	970
	5'-CAGCTCATAGGCCAGTGTCA-3'		
4.	5'-CGATGTGATGGTCAGGATTG-3'	*citD2*, *citE2*	770
	5'-CGGGCGTAGAACAGTTCAGT-3'		
5.	5'-CATCGATGTGATTCGTCAGG-3'	*citF2*, *citG2*	873
	5'-GCAATCAGCTCATCGTCAAA-3'		

**Table 2:** Detection of the 13-kb genomic region in 187 *K. pneumoniae *isolates.

Specimen type (no. of isolates)	Primer 1 *citA*, *citB*	Primer 2 *oadA*, *oadB*	Primer 3 *citS*, *citC2*	Primer 4 *citD2*, *citE2*	Primer 5 *citF2*, *citG2*	Positive*
Urine (93)	56	80	56	58	55	55 (59%)
Non-urine (94)	54	82	54	54	54	53 (56.3%)
Blood (28)	18	25	18	18	18	18 (64.2%)
Wound (23)	11	18	12	12	12	11 (47.8%)
Respiratory (23)	12	20	11	11	11	11 (47.8%)
Other (20)	13	19	13	13	13	13 (65%)

* Note: Strains with positive results using all 5 primer sets are regarded as positive for the 13-kb region.

In uropathogenetic *E. coli *strains, adhesins enable the anchorage to urinary tract to overcome the hydrodynamics of micturition, even though *E. coli *cannot live solely on citrate in anaerobic condition [2]. Other factors in the *K. pneumoniae *genome likely also contribute to urinary infection. To investigate the host-microbial interaction in UTI and to overcome the complex clinical situations, animal models will be necessary for determining the role of this 13-kb genomic island in *K. pneumoniae *in colonizing the urinary tract.

### Genomic diversity on citrate fermentation

The genes associated with citrate fermentation are different in composition and order in the sequenced *Enterobacteriaceae *genomes (Figure 1). In *Salmonella enterica *serovar Typhimurium LT2 (GenBank: AE006468), which is capable of citrate fermentation using the same pathway, two gene clusters similar to the 13-kb region are present in the genome (Figure 1b). One of them (locus I) showing similar gene arrangement (*citAB*, and divergent *citCDEFXGT*) was identified between the *rna *RNase I gene (Locus_tag: STM0617, location: 679989-680795) and the *dcuC *C4-dicarboxylate transporter gene (Locus_tag: STM0627, location: 690391-691776) in the LT2 genome. The other (locus II) (*citS-oadGAB-citAB*, and divergent *citC2D2E2F2X2G2*) was found between *rihC *putative nucleotide hydrolase gene (Locus_tag: STM0051, location: 60164-61084) and *dapB *(Locus_tag: STM0064, location: 74017-74838). Both loci in LT2 carry the *citX *gene in respect to that of the 13-kb island of *K. pneumoniae*. Based on the composition of the gene clusters and the genes at the vicinity, it appears that the second copy (locus II) from LT2 is more related (closer) to the 13-kb island of *K. pneumoniae*, albeit three hypothetical orfs (Figure 1a) next to the *citB *in *K. pneumoniae *are missing in LT2. The first copy of the gene cluster from LT2, as shown in Figure 1b, is similar in gene organization to the citrate fermentation gene cluster in *E. coli *K12 (GenBank: U00096), which contains a *citAB *and a divergent *citCDEFXGT *positioned next to the *rna *RNase I gene (Locus_tag: b0611, location: 643420-644226) (Figure 1c). The *citT *encodes a citrate-succinate antiporter for citrate uptake in *E. coli *[19]. While the citrate fermentation genes corresponding to locus I is missing in *K. pneumoniae*, homologs of the *rna *and *dcuC *identified at the two ends of this gene cluster were juxtaposed to each other in the *K. pneumoniae *NTUH-K2044 (KP1607 and KP1608, location: 1551149-1553412), MGH 78578 (location: 742196-744459) and 342 (location: 2962203-3964466). On the other hand, homologs of the *rihC *and *dapB*, the genes flanking the two ends of the 13-kb genomic island from *K. pneumoniae*, were found adjacent to each other in the *E. coli *K12 genome (Locus_tag: b0030 and b0031, location: 27293-29295).

In the MGH 78578, three *oad *gene clusters were found, one located in the 13-kb citrate fermentation gene cluster, another located at the downstream of the *galETKM *genes for galactose metabolism, and the third located near the *ttdA *and *ttdB *genes for tartrate fermentation [20]. In *K. pneumoniae *342 (GenBank: CP000964), the *oad *gene downstream of *galETKM *is missing while the other two copies were kept. In NTUH-K2044, the *oad(dco) *genes associated with the 13-kb region as well as the other copy proximal to the galactose metabolism genes were missing; only the copy near the tartrate dehydratase genes was found in the genome. As demonstrated in *S. enterica*, oxaloacetate decarboxylase is involved in the fermentation of tartrate, presumably following the reaction of tartrate dehydratase, in which tartrate is converted to oxaloacetate [2,20]. It is conceivable that the *oad *genes were recruited to the vicinity of these genes and evolved into operons dedicated to different metabolic functions. Incorporation of the *oadGAB(dcoCAB) *genes in the 13-kb region is likely a result of a secondary insertion event after the acquisition of the *cit *genes in the ancestral microorganism. This is supported by the data that the G+C contents of the *oad(dco) *genes are apparently higher than the neighbouring orfs (Figure 1).

## Conclusion

This is the first report distinguishing citrate fermentation biotypes of *K. pneumoniae*. It appears that the genomic variation of citrate fermentation genes among these strains might be more extensive than previously thought since only half of the *K. pneumoniae *clinical isolates we tested carry the 13-kb genomic island for citrate fermentation. The possession of these genes contributes to their adaptation to different nutrient conditions.

## Methods

### Klebsiella pneumoniae strains

Eight *K. pneumoniae *NK strains (NK3, NK5, NK6, NK8, NK9, NK25, NK29, and NK245) were collected from the Department of Pathology, National Cheng Kung University (NCKU) Hospital, Tainan, Taiwan [21,22]. Nine CMK strains (CMKa01 through 08, and CMKa10) were collected from Chung Shan Medical University Hospital, Taichung, Taiwan. The *K. pneumoniae *strain CG43 was isolated from Chang Gung Hospital, Taoyuan, Taiwan [23]. Strain NTUH-K2044 was isolated from National Taiwan University Hospital, Taipei, Taiwan [12]. The 188 *K. pneumoniae *strains used to test the association between the citrate fermentation genes and the sites of infection were randomly selected from a nationwide surveillance of antimicrobial resistance collection (Taiwan Surveillance of Antimicrobial Resistance, TSAR) [24]. These clinical strains were not epidemiologically linked. Species identification of the isolates was confirmed by the conventional biochemical reactions [25] in addition to using Vitek Gram Negative Plus Identification card (bioMeìrieux Vitek, Inc. Hazelwood, MO, USA).

### Culture of bacteria

Artificial urine medium (AUM) used in this study was prepared as previously described [15]. Anaerobic cultivations of the bacteria in AUM were performed at 37°C using GasPak™ EZ Gas Generating Pouch Systems (BD, Franklin Lakes, NJ, USA). GasPak™ Dry Anaerobic Indicator Strips were used to assure anaerobic condition (BD, Franklin Lakes, NJ, USA). Overnight liquid culture of the bacterial strains was harvested and washed by AUM using mini centrifuge, then serial-diluted to an initial optical density at 600 nm (OD_600_) of approximately 0.0005 (10,000~20,000× dilution) in AUM. Turbidity of the cultured bacteria was monitored spectrophotometrically at 600 nm.

### Gene disruption of the 13-kb genomic cluster

Disruption of the *citS *together with the nearby regulatory region between the two divergently positioned operons in NK8 genome was done by a method facilitated by λ Red recombinase carried on pKD20 [26]. Two PCR primers (cits-HF: 5'-TTAAATCATC ATGCCGAACA CGATGCTGGC GATGACCAGA TTCCGGGGAT CCGTCGACC-3', citc-HR: 5'-TTTTTTAGCG CTTCGTCATT TCAAAACGAA CTGTATTTCT GTAGGCTGGA GCTGCTTC-3') were used to amplify an aac(3)IV (Apra^R^) apramycin resistance gene from pIJ773 [27] while creating the flanking homologous sequence for recombination. As a result, 39-bp from the left end of the *citS *to the beginning of the *citC2 *(corresponding to location 34604-36125 of the MGH 78578) were disrupted by the apramycin resistant gene in NK8. The gene disruption was confirmed by PCR and DNA sequencing of the corresponding genomic region.

### Detection of citrate fermentation genes

Comparative genomic hybridization (CGH) array (NimbleGen Systems, WI, USA) with probes designed according to the predicted coding sequences spanning the 13-kb genomic region of the *K. pneumoniae *strain NK8 (with 99% sequence identity in average compared to syntenic region of MGH 78578) was used to detect differences of this genomic region among the *K. pneumoniae *clinical isolates. A total of 687 probes were designed isothermally (Tm-balanced) with NimbleGen algorithms across these concatenated CDSs sequences in length of 50-mer with 33-nucleotide overlap between adjacent probe sequences. An intact ribosomal RNA gene cluster (containing 16S-23S-5S rRNAs) was included as a positive control. DNA labelling and hybridization methods of genomic DNA, and signal scanning procedure were performed according to manufacturer's instructions. PCR detections of citrate fermentation genes among other clinical isolates were performed using specific primers listed in Table 1 following standard protocols.

### DNA sequence

The complete genomic sequence of *K. pneumoniae *strain NTUH-K2044 has been deposited to the GenBank (accession no. AP006725)[12]. A fosmid clone, KPA-F06C06, containing the 13-kb citrate fermentation gene region, was selected from a fosmid library of *K. pneumoniae *strain NK8.

## Authors' contributions

YTC, TLL^3^, and SFT drafted the manuscript. YTC, and TLL designed and carried out the functional analyses. KMW, TLL^1^, YML, and HYS performed closure/finishing of the genome sequence. TLL^3^, IWH, JJY, MCL, YCL and JTW collected and classified the bacterial strains. YTC, TLL^1^, KMW, TLL^3^, and SFT analyzed the data. TLL^3^, JJY, MCL, YCL, IJH, JTW, and SFT contributed reagents/materials/analysis tools and participated in design and coordination of the study. YTC, KMW, HYS, and SFT performed annotation. All authors have read and approved the final manuscript.
